# Antioxidant Approaches to Management of Ionizing Irradiation Injury

**DOI:** 10.3390/antiox4010082

**Published:** 2015-01-23

**Authors:** Joel Greenberger, Valerian Kagan, Hulya Bayir, Peter Wipf, Michael Epperly

**Affiliations:** 1 Department of Radiation Oncology, University of Pittsburgh Cancer Institute, 5150 Centre Avenue, Rm. 533, Pittsburgh, PA 15232, USA; E-Mail: epperlymw@upmc.edu; 2 Department of Environmental/Occupational Health, University of Pittsburgh, Pittsburgh, PA 15219, USA; E-Mail: kagan@pitt.edu; 3 Department of Critical Care Medicine, Children’s Hospital of Pittsburgh, Pittsburgh, PA 15224, USA; E-Mail: hub22@pitt.edu; 4 Department of Chemistry, University of Pittsburgh, Pittsburgh, PA 15260, USA; E-Mail: pwipf@pitt.edu; 5 Department of Pharmaceutical Sciences, University of Pittsburgh, Pittsburgh, PA 15260, USA; 6 Department of Bioengineering, University of Pittsburgh, Pittsburgh, PA 15260, USA

**Keywords:** ionizing irradiation, antioxidants, oxidative stress, mitochondrial mechanisms of apoptosis

## Abstract

Ionizing irradiation induces acute and chronic injury to tissues and organs. Applications of antioxidant therapies for the management of ionizing irradiation injury fall into three categories: (1) radiation counter measures against total or partial body irradiation; (2) normal tissue protection against acute organ specific ionizing irradiation injury; and (3) prevention of chronic/late radiation tissue and organ injury. The development of antioxidant therapies to ameliorate ionizing irradiation injury began with initial studies on gene therapy using Manganese Superoxide Dismutase (MnSOD) transgene approaches and evolved into applications of small molecule radiation protectors and mitigators. The understanding of the multiple steps in ionizing radiation-induced cellular, tissue, and organ injury, as well as total body effects is required to optimize the use of antioxidant therapies, and to sequence such approaches with targeted therapies for the multiple steps in the irradiation damage response.

## 1. Introduction

Ionizing irradiation induces sequential steps of cellular, tissue, organ, and total body injury [1,2,3,4,5,6,7,8,9,10,11,12,13,14,15]. Within fractions of a second during ionizing irradiation exposure, water in cells is hydrolyzed and free radicals are generated, including superoxide and hydroxyl radical, which are detectable in experiments [16,17,18,19,20,21,22,23,24,25,26,27,28]. Sequential steps involve the generation of hydrogen peroxide [29]. At this stage (seconds), and for minutes, hours, and days after exposure, the management of oxidative stress from ionizing irradiation shares many steps common with other forms of injury, including: Hyperbaric oxygen injury, hypoxia, chemical toxin exposure, heat, ultraviolet irradiation, chemical toxicity, and infection [30,31,32,33]. Over days after irradiation, tissues and organs respond to a sequence of events culminating in an acute inflammatory response [1,30,31,34,35,36,37,38,39,40,41,42,43,44]. Somewhat unique to radiation injury is the subsequent latent period during which many injury markers, including histopathologic effects are undetectable [32,45,46,47,48,49,50,51,52,53,54,55]. Tissue volume, tissue type, and genetic factors control the onset of a final late/chronic radiation injury phase, which includes fibrosis and scarring [48].

**Figure 1:**
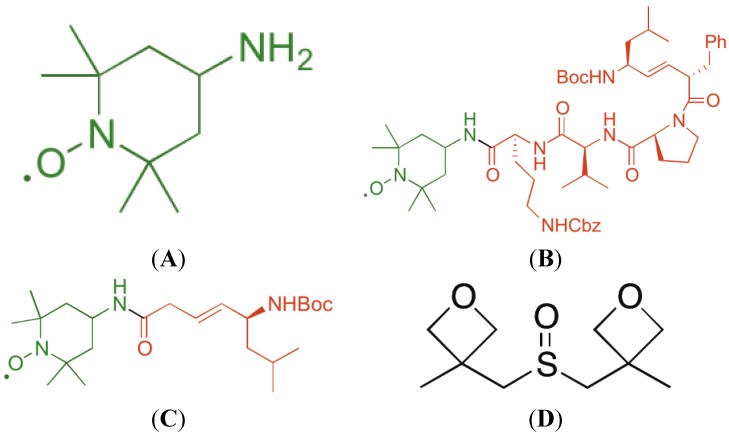
Structures of 4-Amino-Tempo (**A**); XJB-5-131 (**B**); JP4-039 (**C**); and MMS350 (**D**).

In recent years, attempts to ameliorate ionizing irradiation injury have utilized reagents and therapeutics common to the treatment of other forms of tissue and organ injury [56]. Prominent among these attempts has been the approach to utilize antioxidant therapies. Initial experiments using free radical scavengers include *N*-Acetyl-Cysteine (NAC) or the supplement/replenishing of antioxidant stores by delivering glutathione. These methods demonstrated some success in tissue culture and animal models [57,58,59,60,61,62]. However, true breakthroughs occurred with the molecular cloning and expression of transgenes for antioxidant enzymes, prominently the superoxide dismutases [63,64,65,66,67]. Replenishment of superoxide enzymes by protein delivery was ineffective compared to delivery of transgene for protein production [63,64]. The relatively cumbersome nature of producing transgenes for gene therapy invigorated the development of small molecule antioxidants with properties similar to the transgene products. Prominent in this category was the development of Tempo (Figure 1), a nitroxide with the capacity to scavenge free radicals and, through cycling of the nitroxide to hydroxyl-amine, to neutralize multiple free radicals for each molecule of Tempo [59,68]. Finally, the role of mitochondria in the mechanism of irradiation apoptosis prompted the development of mitochondrial-targeted Tempo (GS-nitroxides and triphenylphosponium nitroxides) [29,68,69,70,71,72,73,74]. Initial success in animal models using mitochondrial-targeted antioxidants has uncovered multiple steps in the response to ionizing irradiation, which cannot be explained solely by individual cellular responses (nuclear-mitochondrial signaling and apoptosis). These realizations have led to new therapeutic approaches including the application of new drugs, with actions distinct from the antioxidant approach. Nevertheless, three categories of applications of mitochondrial-targeted antioxidants have gained prominence as potential therapeutic strategies toward the amelioration of irradiation injury.

## 2. Experimental Section

### 2.1. Mice

The C57BL/6NHsd, FVB/N, 129/Sv, and Fanconi Anemia (FA) Fancd2^−/−^ mice on both the 129/Sv and C567BL/6 genetic backgrounds have been described previously [75,76]. Mice were housed four per cage according to the Institutional IACUC Protocol, and fed standard laboratory chow and deionized water.

### 2.2. Tissue Culture Experiments

Techniques for long-term bone marrow culture, establishment of bone marrow stromal cell lines, and Interleukin 3 (IL-3) dependent hematopoietic progenitor cell lines have been published [36,77]. Methods for growing fresh bone marrow colony forming units CFU-GEMM have been published previously [78].

### 2.3. Manganese Superoxide Dismutase-Plasmid Liposomes

The techniques for production and administration of MnSOD-PL have been described previously [1,26]. Briefly, the MnSOD transgene was expressed in plasmid vector, the plasmid was grown according to published methods, and it was delivered in a liposome preparation of cationic liposomes by an intra-tracheal installation [1,2,3], intravenous route [79,80,81], intra-oral [40], or intraesophageal [6,82] route according to previous publications.

### 2.4. GS-Nitroxides and JP4-039

**Figure 2:**
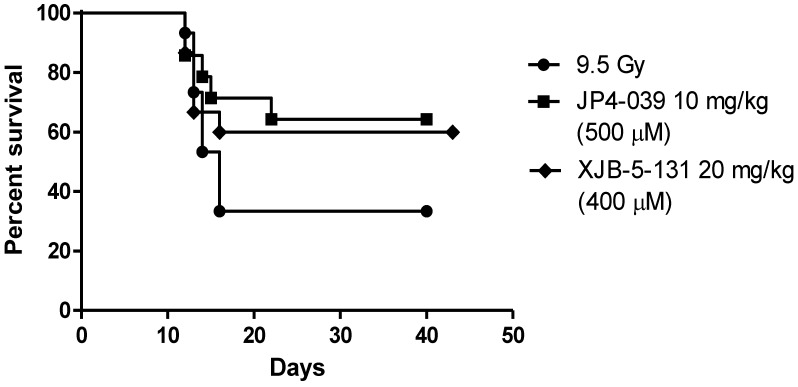
Effective radiation mitigation by two GS-nitroxide analogs (XJB-5-131 and JP4-039). Groups of 15 C57BL/6NHsd mice received total body irradiation, and then 24 h later intravenous administration of 100 μL of F14 liposomes containing either XJB-5-131 or JP4-039, standardized for equimolar concentration. The heavier molecular weight of XJB-5-131 requires larger quantities to achieve an equimolar concentration with JP4-039. There was equivalent radiation mitigation by both drugs.

**Figure 3:**
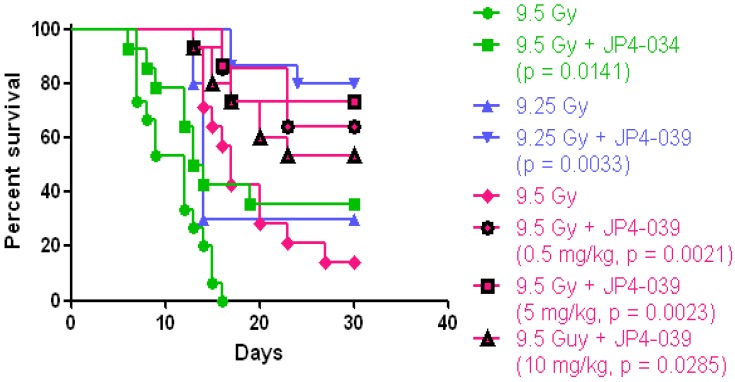
Effective mitigation of total body irradiation damage in C57BL/6NHsd mice by intravenous administration of JP4-039. Experiments shown are over a course of a year accounting for the TBI dose “drift” of the LD_50/30_. At 3 different time points during a single calendar year, experiments were carried out delivering JP4-039/F15 in 100 μL volume containing 20 mg/kg drug, to mice. In these experiments, the LD_50/30_ was noted to “drift” over the course of the year and could not be explained by changes in the Cesium-70 Gamma Cell irradiator, supplier of mice, age of mice, gender of mice (all were female), diet, or other factors in the animal care facility. In all experiments, despite the “drift” of the LD_50/30_, JP4-039 was an effective mitigator against total body irradiation.

**Figure 4:**
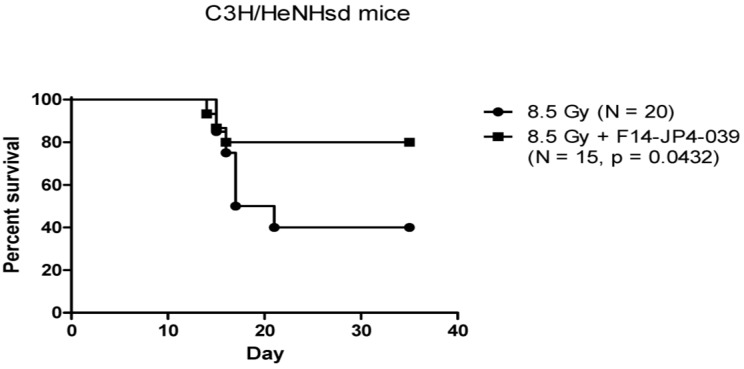
Mitigation of total body irradiation damage to C3H/HeNHsd mice by intravenous JP4-039/F14 antioxidant small molecule therapy. Groups of C3H/HeNHsd mice (*n* = 15) received total body irradiation, and 24 h later intravenous administration of 100 μL of F14 liposomes containing 100 μg of JP4-039. Survival was quantitated, and there was a significant increase in survival in mice given JP4-039.

The mitochondrial-targeted nitroxide based on 4-Amino-Tempo (Figure 1) have been described in detail [42,68,83,84]. GS-nitroxide drugs were prepared by attachment of modified gramicidin S fragments to the nitroxide, thus generating mitochondrial targeted agents of various lengths (Figure 1A–C). These included the larger molecule XJB-5-131 [85], and a shorter analog, JP4-039, which have been shown to be radiation mitigators (Figure 2 and Figure 3) [68]. JP4-039 has been demonstrated to be effective as a total body radiation protector and mitigator [80,86] in both C57BL/6NHsd mice (Figure 3) and in C3H/HeN mice (Figure 4). The laboratory of Dr. Peter Wipf of the Department of Chemistry at the University of Pittsburgh synthesized XJB-5-131, JP4-039 as well as MMS350, a highly water soluble sulfoxide with a different mechanism of action for prevention of radiation damage.

### 2.5. Assays for Antioxidant Stores

The Trolox assay for antioxidant stores has been published in detail and these methods have been previously described [75,76].

### 2.6. Assays for Apoptosis, Mitochondrial Content, and Mitochondrial Number

The methods for quantitation of ionizing irradiation effects on mitochondria have been published previously [28,87].

## 3. Results and Discussion

### 3.1. Antioxidant Therapies to Prevent and/or Mitigate Total Body Irradiation Injury

Two applications of antioxidant therapies in ionizing irradiation damage have recently been described. Protection against total body irradiation damage has been demonstrated with MnSOD-plasmid liposomes administered intravenously 24 h prior to total body irradiation [79,81]. In two model systems, one in which MnSOD-PL was given alone [79], and in another system supplemented with an antioxidant diet delivered after irradiation [81], improved survival of both male and female mice was demonstrated. The 24 h time point before irradiation was chosen based on previous studies that demonstrated a requirement for this time to get transgene into cells [66,88,89,90,91,92,93], facilitate nuclear migration, insertion of the plasmid into the nucleus, production of RNA for MnSOD, production of MnSOD, and then transport of the mitochondrial targeted SOD to the mitochondria, where radiation protection and mitigation actions were demonstrated [77,94,95,96]. Tissue culture studies demonstrated the requirement for mitochondrial targeting. In initial studies, cytoplasmic SOD1 (Cu/ZnSOD) was demonstrated to have little radioprotective or mitigation effect. However, when the mitochondrial targeting sequence from SOD2 (MnSOD) was added to Cu/ZnSOD, the molecule targeted the mitochondria and was radiation protective [36,49]. In contrast, when the mitochondrial targeting sequence was removed from the MnSOD transgene product, little radiation protection was seen, and gene product was concentrated in the cellular cytoplasm [49].

Antioxidant therapies to prevent total body irradiation damage were extended by the development of small molecule SOD mimics [17,18,19,42,70,97,98,99,100,101]. One strategy utilized in our laboratory was to increase the effectiveness of the nitroxide 4-Amino-Tempo (4-AT) by facilitating a mitochondrial enrichment [68]. Several GS-nitroxide variants were developed in the laboratory of Peter Wipf, Ph.D. [42,102,103]. Mitochondrial targeting was achieved by using a hemigramicidin analog attached to 4-Amino-Tempo [68]. Two different GS-nitroxides were compared for *in vivo* radiation mitigation, when delivered 24 h after total body irradiation. Figure 2 demonstrates the similar effectiveness of XJB-5-131, which shows a 300–600-fold mitochondrial concentration capacity, specifically in the inner mitochondrial membrane, compared to JP4-039, a molecule with a truncated mitochondrial targeting sequence, and a *ca.* 20-30-fold increased mitochondrial concentration. As shown in Figure 2, both molecules delivered in equimolar concentration 24 h prior to total body irradiation showed significant mitigation capacity.

The difference between radiation protection and mitigation has particular relevance for the Radiation Counter Measures Program of the National Institutes of Allergy and Infectious Disease (NIAID) of the National Institutes of Health (NIH) [89]. Delivery of a potent preventive drug prior to irradiation, which can target mitochondria and prevent the depletion of antioxidant stores has great relevance for first responders in an irradiation incident, where accumulation of radio-isotopes might be expected, or whether further exposure to photon or high linear energy transfer particle (neutron, proton) irradiation might occur. However, the radiation protection strategy is not relevant to victims of a radiation terrorist event or nuclear reactor damage in which irradiation exposure would take place prior to the administration of drugs. Delivery of an irradiation modifying drug 24 h or later after irradiation is considered “mitigation”, and thus the radiation mitigator properties have been a focus of the Radiation Counter Measure Program. Figure 2 demonstrates that both XJB-5-131 and JP4-039 were effective mitigators despite a significant difference in mitochondrial concentration capacity. This data argues that the difference between a 30-fold and 600-fold mitochondrial concentration capacity might not be relevant to a successful total body radiation mitigation. However, other studies have demonstrated the effectiveness of XJB-5-131 in crossing the blood brain barrier to ameliorate the toxicity of traumatic brain injury, while JP4-039 does not cross the blood brain barrier [85].

The mechanism of total body irradiation protection and mitigation by both gene therapy (MnSOD-PL), and small molecule antioxidants (GS-nitroxides) [16,42,57,58,77,79,83,94,95,96,97,104,105,106,107,108] may not be attributable solely to direct cellular effects. The bystander effect of both radiation injury and amelioration of irradiation damage by radiation mitigator drugs has been well documented in the radiobiology literature [76]. Most recently, using Fancd2^−/−^ (129/Sv) mice, a bystander effect of partial body irradiation was demonstrated. Head and neck irradiation of mice demonstrated significant suppression of bone marrow at the distant femur site [76]. Application of a mitochondrial targeted antioxidant radiation protector/mitigator to the oral cavity/oropharynx, to be described in the next section, not only protected the treated tissue, but also suppressed the bystander effect of radiation damage. A positive bystander effect also applies to the application of radiation protectors and mitigators. Uptake and mitochondrial targeting of drug in a relatively small percentage of cells within an organ can confer organ radiation protection or mitigation [1,40,90]. This was demonstrated with esophageal delivery of hemagglutin-epitope tagged MnSOD transgene product, which, when delivered prior to irradiation conferred significant organ radioprotection, while the transgene product was only detectable in a small fraction of cells within that organ [39]. These data were confirmed in both lungs and esophagus as well as oral cavity organ specific radiation protection strategies (These data will be described in the next section regarding acute radiation injury to specific organs and targeted therapies using the antioxidant approach).

### 3.2. Antioxidant Radioprotection and Radiation Mitigation in Organ Specific/Localized Applications

The effectiveness of the antioxidant approach in both preventing and ameliorating irradiation injury was demonstrated in model systems utilizing both MnSOD-PL gene therapy [45], and also more recently with targeted GS-nitroxides [68].

Organ specific radioprotection using MnSOD-PL has been demonstrated in bladder [37], intestine [46], oral cavity/oropharynx [40,41], esophagus [6,109], lung [1,90], and in fetal mice *in vivo* [80]. In each of these systems, MnSOD-PL administration utilized a localized liposomal free system, Herpes Virus vector or adenovirus vector administered transgene. Organ specific radiation protection/mitigation was demonstrated in each model system in mice or rats utilizing radiobiological parameters of injury including histopathology, organ function, physiologic monitors (bladder measurement of water and urea transepithelial transfer), and by assays for specific cellular damage including the apotag assay for apoptosis, and electron microscopic evidence of cell death [17,18,19,59,98,99,110].

Application of GS-nitroxides as organ specific radioprotectors followed the work of MnSOD-PL mediated radiation protection, and utilized the same organ specific radiation damage systems. JP4-039, which demonstrated significant total body irradiation protection and mitigation properties (described in the last section) was highly effective when delivered in formulations designed to keep drug localized to a specific organ system [76]. JP4-039 was delivered in a liposomal emulsion containing Tween (F15), which was designed to keep the drug localized and in direct contact with cells exposed to the drug. The JP4-039/F15 formulation was a highly effective radiation protector and mitigator for esophagus and oral cavity/oropharynx [76,111]. In recent studies, JP4-039/F15 administration to both FA Fancd2^−/−^ (129/Sv) and Fancd2^−/−^ (C57BL/6) mice, as well as heterozygote and wild type litter mates showed significant protection and mitigation of damage to the oral cavity/oropharynx in both single fraction and fractionated irradiation [76,112]. In models of organ specific radiation protection/mitigation, principally designed for translational approaches in clinical radiotherapy, there has been great concern that antioxidant drugs would also protect tumors. Initial studies with MnSOD-PL as well as JP4-039/F15 demonstrated no protection of orthotopic lung tumors [9,41] or orthotopic head and neck cancer [96,112].

### 3.3. Antioxidant Therapy to Prevent Ionizing Radiation Late Effects

There are several components to ionizing irradiation-induced late effects [59]. It has been demonstrated that volume of tissue/organ irradiated, total dose of irradiation, and in the case of clinical radiotherapy, the fraction size of radiation, all contribute to the severity and time of onset of late effects [48]. In clinical radiotherapy, late effects are most critical in producing organ failure [79]. Kidney, liver, lung, and esophageal irradiation are known to induce fibrosis, which can limit the effectiveness of treatment due to late side effects [48,50]. In the case of total body irradiation, late effects are most prominent after relatively low doses of irradiation that, while suppressing natural blood counts, do not lead to acute radiation damage. The most prominent late effect is the induction of cancer [79]. The molecular mechanism of irradiation late effects has been the subject of intense investigation. Antioxidant approaches to prevent late effects of irradiation have gained significant interest in recent years. Oxidative stress has been shown to be a chronic component of irradiation damage to tissues and organs. These oxidative stress events are detected in the lung years after therapeutic lung irradiation [57]. In animal models, markers of antioxidant stress are detected in irradiated lungs months after irradiation[54]. A critical approach has been taken to utilize antioxidant therapies to ameliorate late effects. The administration of MnSOD-plasmid liposomes intravenously prior to irradiation has been shown to significantly improve acute survival measured by the lethal dose for 50% of mice at 30 days (LD_50/30_), but also to significantly ameliorate radiation late effects, prominently life shortening and carcinogenesis [79,113]. In a second series of experiments, mice given MnSOD-PL 24 h prior to irradiation were then placed on a novel antioxidant diet consisting of a wide variety of antioxidants and chemopreventive agents [81]. Under these conditions, surviving animals demonstrated significant further amelioration of radiation-induced life shortening. In both of these experiments, MnSOD-PL alone, or supplemented with an antioxidant diet, there was no increase in detectable cancers in mice surviving for prolonged periods after irradiation.

There is much evidence to support a difference in the molecular mechanism of acute irradiation damage compared to late effects [54,55,114]. The administration of radioprotective MnSOD-PL into the lungs prior to irradiation was significant in preventing acute radiation pneumonitis and death. However, in a mouse model that received MnSOD-PL 100 days after irradiation, when late effects (radiation fibrosis) began to appear, late effects were not ameliorated [47]. Studies with pulmonary irradiation in a fibrosis prone mouse strain (C57BL/6NHsd) compared to a fibrosis resistant mouse strain (C3H/HeNHsd) demonstrated a significant difference in radiation responses in the lungs with respect to the RT-PCR detected induction of RNA transcripts [55]. Fibrosis prone C57BL/6NHsd mice showed upregulation of TLR4 (Toll-Like Receptor 4) in the lung at the time of the initiation of fibrosis, while C3H/HeNHsd mice did not show upregulation of TLR4 [115].

Furthermore, the critical role of TGF-β in late irradiation fibrosis has been demonstrated in SMAD3^−/−^ knockout mice [116,117], which demonstrated no irradiation-induced skin fibrosis, and no detectable radiation pulmonary fibrosis. Migration into the lungs of bone marrow stromal cell progenitors of lung fibroblasts has been shown to be a significant component of pulmonary fibrosis following irradiation. In an experiment in which wild type mice were chimeric for either SMAD3^−/−^ fluorochrome labeled bone marrow stromal cells or wild type sex mismatched fluorochrome labeled bone marrow stromal cells, there were significant decreases in migratory capacity of the SMAD3^−/−^ stromal cells into the lung contributing to radiation fibrosis [117]. Decreased motility of bone marrow stromal cells from SMAD3^−/−^ mice was also characteristic of this genotype, suggesting further contribution to the fibrotic phenotype after irradiation of the motility of the stromal cells [116].

A recent advance in prevention of irradiation late effects has been the demonstration of a water-soluble dimethylsulfoxide analog, MMS350 (Figure 1), which, when administered to mice beginning 100 days after thoracic irradiation, significantly ameliorated fibrosis [54]. MMS350 has been shown to be easily administered in mL drinking water and is safely delivered to mice continuously after thoracic irradiation.

Effects of irradiation have been shown to be common to other forms of toxic substance induced fibrosis. In recent studies of liver fibrosis, the involvement of specific cytokine receptors has been shown to be mediated at the level of the endothelial cells [118,119]. Involvement of endothelial cells in radiation fibrosis is also supported by studies with the von Willebrand Factor (vWF^−/−^ knockout mice), which showed reduced fibrosis [120]. TLR4^−/−^ (knockout) mice also showed reduced radiation fibrosis [115]. While the molecular biologic response is complex, endothelial cells appear to be gate-keepers for the signaling determining whether tissues repair radiation damage by restoration of tissue function or demonstrate a damage signal that solicits migration from the bone marrow into that organ of bone marrow stromal cell progenitors of fibrosis and induces the late fibrotic phenotype [110].

## 4. Conclusions

There is much evidence to support an oxidative stress model for both acute ionizing irradiation effects and chronic oxidative stress mediated irradiation late effects. The initiation of therapeutic programs to combat radiation damage began with studies of transgene therapy utilizing mitochondrial targeted MnSOD-Plasmid Liposomes. These have been demonstrated in a recent clinical trial to be safe and effective in ameliorating acute irradiation toxicity to the esophagus [121]. MnSOD-PL therapy was shown to be effective in preventing radiation damage in total body experiments, and also in organ specific irradiation. The translation of this technology into clinical radiotherapy is in progress. A more recent development has been the use of MnSOD-mimic compounds and unnatural anti-antioxidants. XJB-5-131 and JP4-039 are enriched in mitochondria, and, consequently, seem to be particularly effective [122,123]. Triphenylphosphonium targeted nitroxides are also effective radiation protectors and mitigators [124]. Most recently, a highly water-soluble dimethylsulfoxide analog, MMS350, has been shown to be safe when administered in drinking water and prevents both the acute and chronic effects of total body irradiation or pulmonary irradiation [54]. Further studies will be required to optimize the delivery of specific agents designed to prevent irradiation-induced apoptosis. Perhaps even more important is the mounting evidence that other forms of irradiation damage are involved in cellular, tissue, and organ responses to radiation, including necroptosis, ferroptosis, and alteration in oxidative lipidomics of tissues [43,71,101]. This damage leads not only to localized tissue injury, but also effects migration into tissues of inflammatory cells, which, through the production of inflammatory mediators, exacerbate the radiation response. Oxidative stress remains a prominent factor in ionizing irradiation damage and the use of antioxidants in both the fundamental study of radiation countermeasures and in clinical radiotherapy appears to be warranted.
